# New Projectile

**Published:** 1868-02

**Authors:** 


*New Projectile.*—The rapid advance of the military nations in the improvement of fire-arms, has led to improved forms of projectiles. It has been discovered, as might have been anticipated, that a rapidly travelling missile is apt at a certain distance, to make a clean cut through the soft parts. The old writers on military surgery tell us that the aperture of exit is always greater than that of entrance. However true this may be of the round ball, it is not invariably true of the conical bullet. The irregularity of the wound made by the modern missile appears to depend very greatly upon the amount of shattering it has undergone. Driven at high velocity at short range, say one hundred yards, and penetrating an object which offers considerable resistance, its form will be greatly changed and its track very irregular and jagged. At a lower velocity, it will penetrate deeper, come out comparatively unaltered, and leave a clear entrance wound. As a rule, the recent wars have given us wounds with clearly cut apertures of entrance and exit.

The Indian tiger-hunters, recognizing this fact, have begun to return to the round bullet as inflicting a more violent wound. Some of them have adopted a short conical bullet with a metallic case charged with percussion powder, inserted into its centre. There is small chance of the tiger continuing his charge when he receives such a missile as this. The explosion of the shell drives the fragments in all directions through the tissues, tearing up the muscles, pulverizing the bones, and producing terrific nervous shock, while the great volume of gas developed by the explosion greatly increases the disorganization. This murderous missile Herr von Dreyse is said to have introduced into the Prussian army, having adapted it to the needle-gun.

The Chassepot bullet, though constructed on a different principle, is claimed to be nearly as effective. This projectile contains a cylindrical cavity bored from the summit of the cone to about two-thirds the length of the ball, and having its upper orifice closed with a plug of boxwood or even beeswax. When the bullet strikes fair upon the head the plug is driven in, forcing the contained air out in all directions, and spattering out the bullet in a very remarkable manner, even to the extent sometimes of driving out its whole base. Of course the laceration is very great.

The savage ingenuity displayed in the construction of these formidable implements of war does not speak much for the persuasive powers of the Peace Society. Surgery, too, in its military department is becoming a much more difficult art, in consequence of the increased destructiveness of fire-arms.

